# Supplementary material for: Microvascular stabilization via blood-brain barrier regulation prevents seizure activity
Source: Nat Commun. 2022 Apr 14;13:2003. doi: 10.1038/s41467-022-29657-y (PMC9010415; doi:10.1038/s41467-022-29657-y)
Supplement: Supplementary file 1 — Supplementary Information [file 41467_2022_29657_MOESM1_ESM.pdf]

Supplementary Information

Microvascular stabilization via blood-brain barrier regulation prevents seizure activity

Chris Greene[1], Nicole Hanley[1], Cristina R. Reschke[2,3], Avril Reddy[1], Maarja A. Mäe[4], Ruairi Connolly[2,5,6], Claire Behan[2,5,6], Eoin O'Keeffe[1], Isobel Bolger[1], Natalie Hudson[1], Conor Delaney[1], Michael A. Farrell[7], Donncha F. O'Brien[8], Alan Beausang[7], Christer Betsholtz[4], David C. Henshall[2,9], Colin P. Doherty[2,5,6] and Matthew Campbell[1,2]*

[1]Smurfit Institute of Genetics, Trinity College Dublin, Dublin 2, Ireland.

[2]FutureNeuro, Science Foundation Ireland Research Centre for Chronic and Rare Neurological Diseases, Royal College of Surgeons in Ireland, University of Medicine and Health Sciences, Dublin, Ireland.

[3]School of Pharmacy and Biomolecular Sciences, RCSI University of Medicine and Health Sciences, Dublin 2, Ireland.

[4]Department of Immunology, Genetics, and Pathology, Rudbeck Laboratory, Uppsala University, Sweden

[5]Department of Neurology, Health Care Centre, Hospital 5, St James's Hospital, Dublin 8, Ireland.

[6]Academic Unit of Neurology, Room 5.41, Biomedical Sciences Institute, Trinity College Dublin, Dublin 2, Ireland.

[7]Department of Neuropathology, Beaumont Hospital, Dublin, Ireland.

[8]Department of Neurosurgery, Beaumont Hospital, Dublin, Ireland.

[9]Department of Physiology and Medical Physics, RCSI University of Medicine and Health Sciences, Dublin 2, Ireland.

*To whom correspondence should be addressed

Dr Matthew Campbell

Phone: 353 1 8961482

Fax: 353 1 8963848

Email: matthew.campbell@tcd.ie

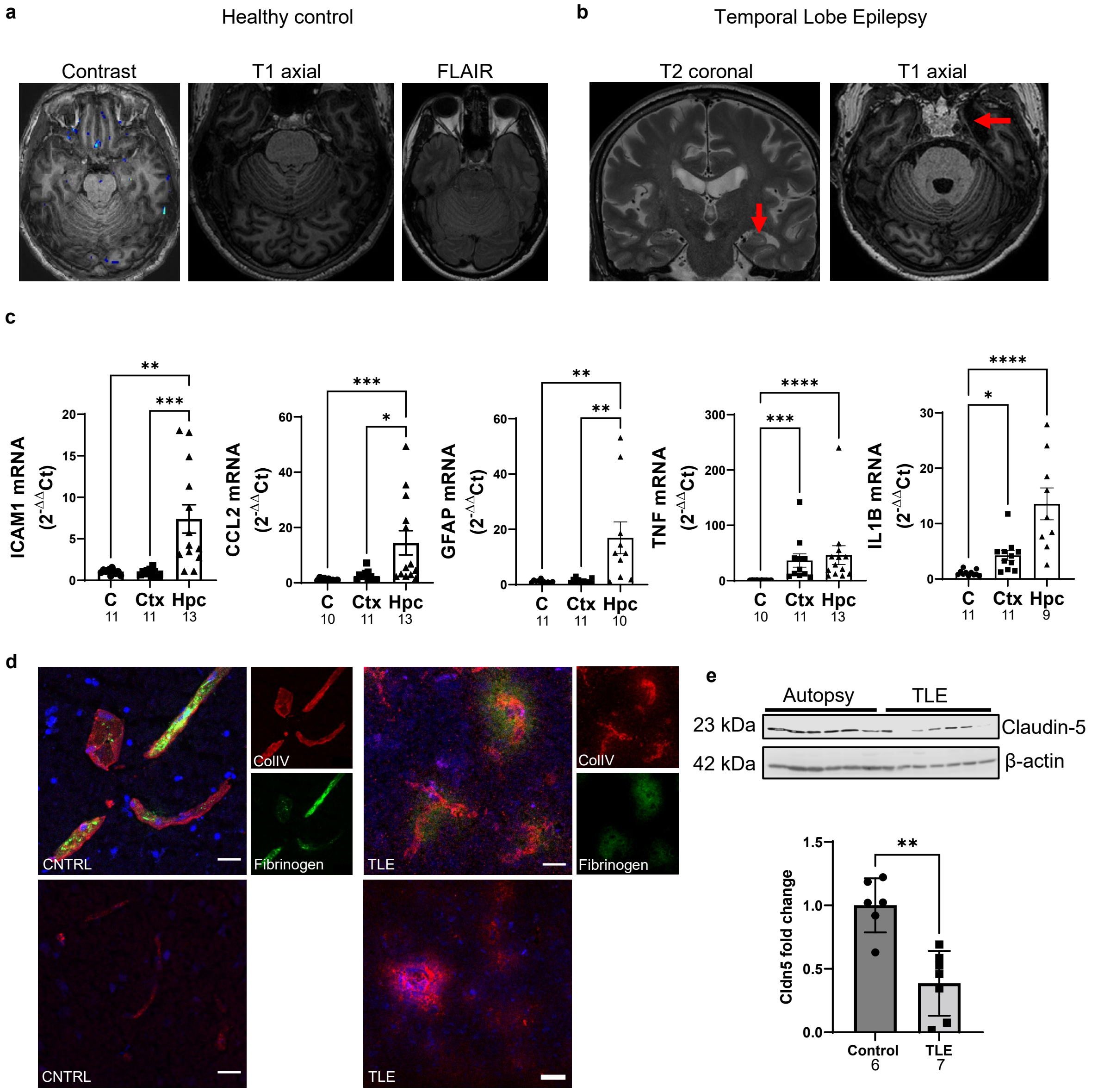

Supplementary Figure 1. Increased neurodegeneration, neuroinflammation and reduced BBB integrity in the temporal lobe of drug resistant temporal lobe epilepsy (TLE) patients. a) T1 with contrast, T1 axial scan and FLAIR scan in a healthy volunteer. **b)** T2 coronal and T1 axial scan in a TLE patient showing hippocampal and temporal lobe atrophy (red arrows). **c)** Increased mRNA expression of ICAM1 (**p=0.0025 Hpc vs C; **p=0.0001 Hpc vs Ctx), CCL2 (**p=0.0001 Hpc vs C; *p=0.0394 Hpc vs Ctx), GFAP (**p=0.0018 Hpc vs C; **p=0.0058 Hpc vs Ctx), TNF (***p=0.0007 Ctx vs C; ****p<0.0001 Hpc vs C) and IL1B (*p=0.0158 Ctx vs C; ****p<0.0001 Hpc vs C) in resected tissue from the cortex (Ctx) and hippocampus (Hpc) of TLE patients compared to autopsy controls (C). **d)** Collagen IV (red) and fibrinogen (green) staining in control and TLE brain (top). Albumin (red) staining in control and TLE brain (bottom). Images are representative of 3 control and 4 TLE brains. Scale

bars, 20 μm. **e)** Western blot analysis of claudin-5 protein expression in the hippocampus in autopsy control and TLE patient brain (**p=0.0023). Data represent means ± s.e.m.; each data point represents one patient. Number of subjects (n) is indicated on graphs. Kruskal-Wallis test followed by Dunn's multiple comparison test for human gene expression data. Mann-Whitney test for Western blot analysis. *p<0.05; **p<0.01; ***p<0.001; ****p<0.0001. Source data are provided as a Source Data file.

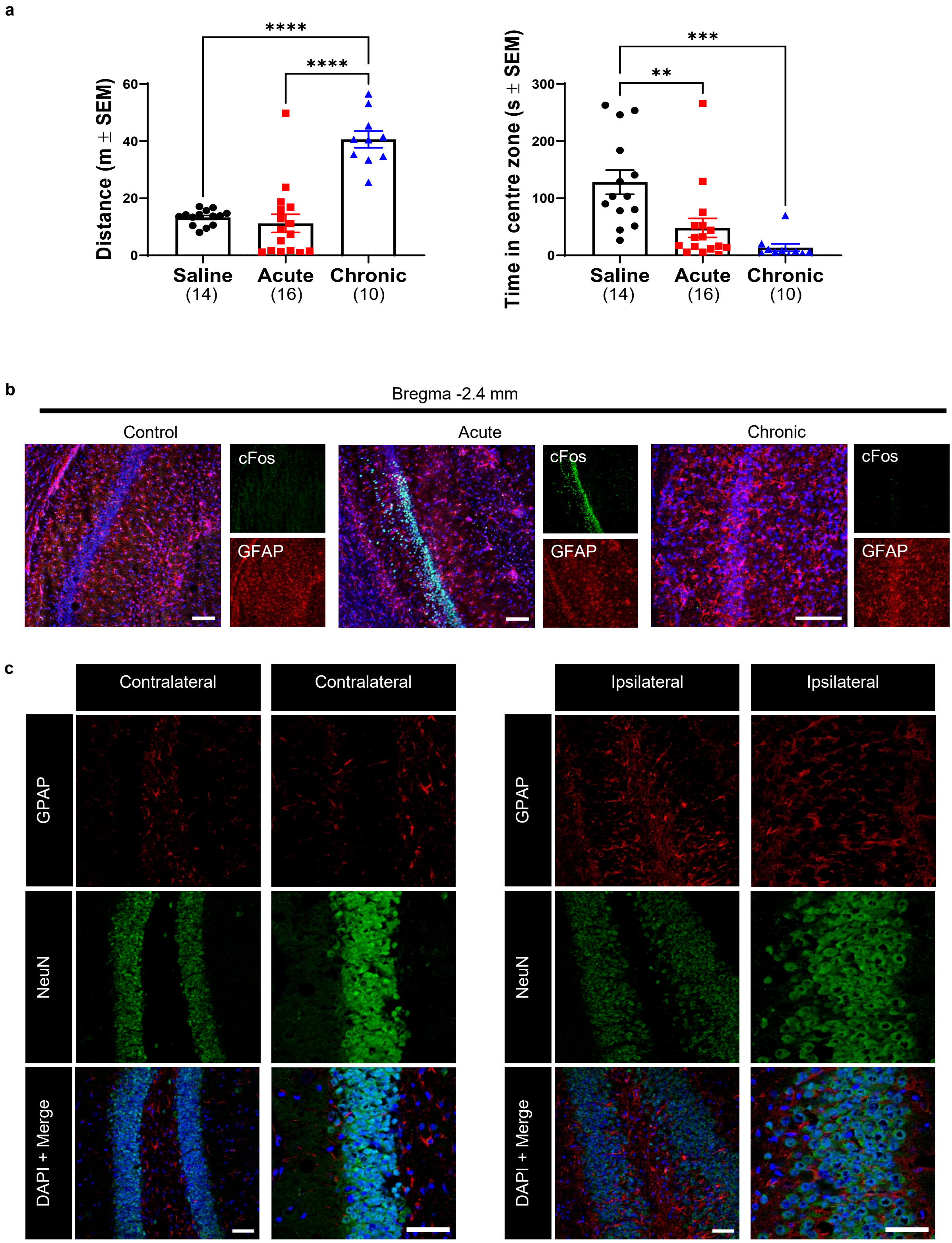

Supplementary Figure 2. Behavioural phenotyping following intrahippocampal injection of kainic acid. a) Distance travelled (****p<0.0001 Chronic vs Saline; ****P<0.0001 Chronic vs Acute) and time spent in the centre zone of the open field test (**p=0.0044 Saline vs Acute; ***p=0.0003 Saline vs Chronic) following intrahippocampal injection of 0.3 µg kainic acid.

b) GFAP (red) and cFos (green) in the hippocampus CA1 region at acute and chronic time points following intrahippocampal injection of 0.3 µg kainic acid. Scale bar, 100 µm. **c)** GFAP (red) and neun (green) in the hippocampal dentate gyrus and CA1 at chronic time points following intrahippocampal injection of kainic acid. Scale bars, 100 µm for 20X images; 50 µm for 40X images. Data represent means ± s.e.m; each data point represents one animal. Number of animals (n) is indicated on graphs. Groups were compared by one-way ANOVA followed by Tukey's multiple comparison test. Images from **b)** and **c)** are representative of n=3 animals per group. **p<0.01; ****p<0.0001. Source data are provided as a Source Data file.

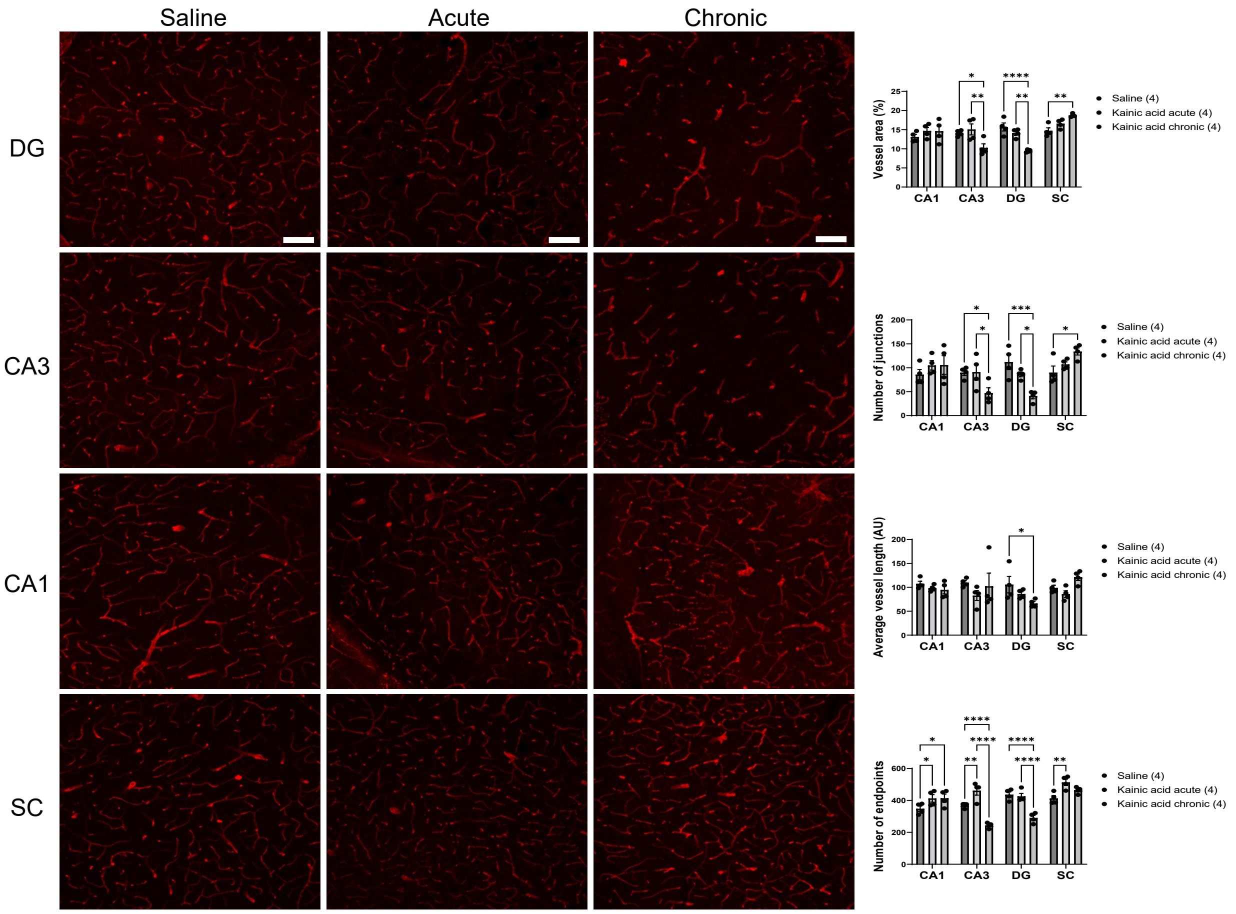

Supplementary Figure 3. Acute and chronic vascular remodelling following intrahippocampal injection of kainic acid. Collagen IV staining (red) in saline injected and kainic acid injected mice at acute and chronic timepoints (left) following intrahippocampal injection of 0.3 µg kainic acid. Vessel area was significantly decreased in the CA3 (*p=0.0107) and dentate gyrus (DG) (****p<0.0001) and significantly increased in the somatosensory cortex (SC) (**p=0.0069) at the chronic timepoint. Number of junctions was significantly decreased in the CA3 (*p=0.0345) and DG (****p<0.0003) and significantly increased in the SC (**p=0.0288) at the chronic timepoint. Average vessel length was significantly reduced in the DG (*p=0.0405) at the chronic timepoint. Number of endpoints was significantly increased in the CA1 at acute (*p=0.0463) and chronic (*p=0.0416) timepoints; in the CA3 at acute (**p=0.0032) only and in the SC at acute (**p=0.0014). Endpoints were decreased in the CA3 (****p<0.0001) and DG (****p<0.0001) at chronic timepoints. Data represent means ± s.e.m; each data point represents one animal. Number of animals (n) is indicated on graphs. Groups were compared by one-way ANOVA followed by Tukey's multiple comparison test. Scale bars, 50 µm. *p<0.05; **p<0.01; ***p<0.001; ****p<0.0001. Source data are provided as a Source Data file.

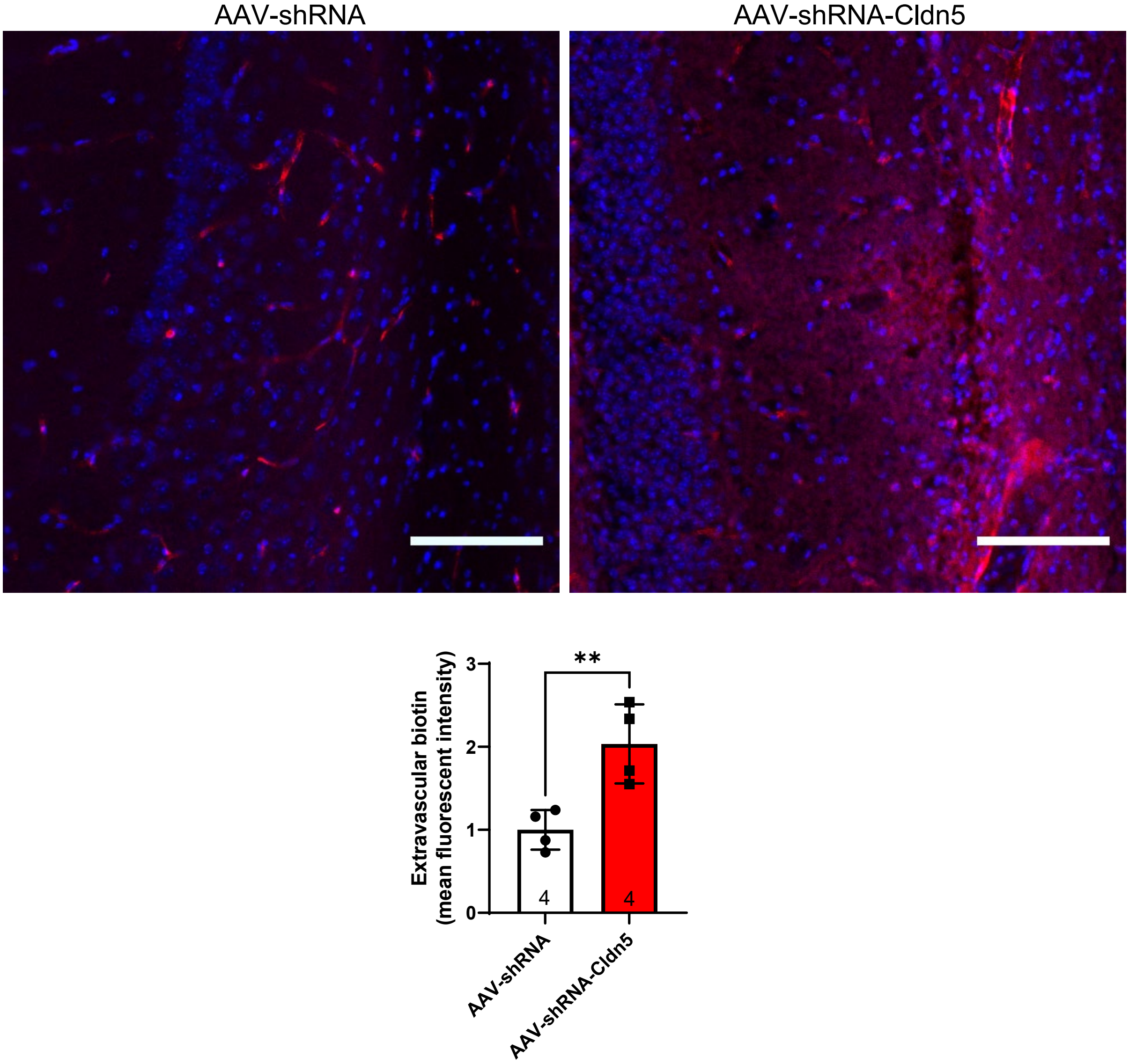

Supplementary Figure 4. Increased BBB leaking to Sulfo-NHS-Biotin following intrahippocampal injection of AAV-shRNA-Cldn5. Biotin staining in AAV-shRNA and AAV-shRNA-Cldn5 injected mice and quantification (**p=0.0082). Data represent means ± s.e.m; each data point represents one animal. Number of animals (n) is indicated on graphs. Groups were compared by two-sided unpaired *t*-test. Scale bar, 50 µm. **p<0.01. Source data are provided as a Source Data file.

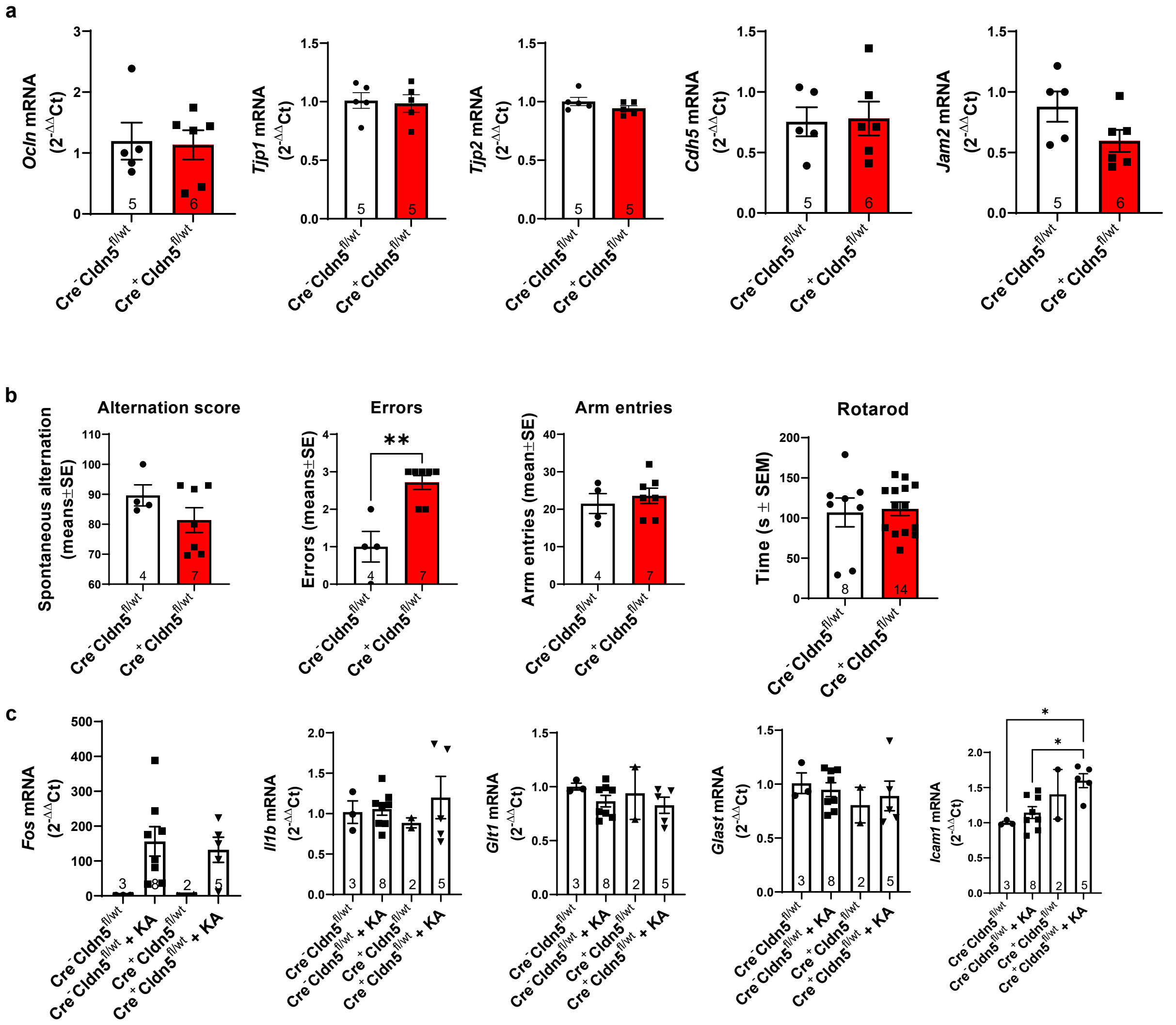

Supplementary Figure 5. Characterisation of Cldn5$^{Fl/wt}$ mice. a) No change in the expression of *Ocln*, *Tjp1*, *Tjp2*, *Cdh5* and *Jam2* gene expression in *Cre$^-$Cldn5$^{fl/wt}$* and *Cre$^+$Cldn5$^{fl/wt}$* mice. **b**) Spontaneous alternation in the y-maze. Spontaneous alternation errors were significantly increased in *Cre$^+$Cldn5$^{fl/wt}$* mice (**p=0.0016). Total arm entries in the y-maze. Time spent on the rotarod. **c**) *Fos*, *Il1b*, *Slc1a2*, *Slc1a3* and *Icam1* (*p=0.0272) mRNA expression 3 hours following i.p. injection of 15 mg/kg kainic acid. Data represent means ± s.e.m; each data point represents one animal. Number of animals (n) is indicated on graphs. Two-sided unpaired *t*-test for (a-b); one-way ANOVA followed by Tukey's multiple comparison test for all other graphs. *p<0.05, **P<0.01. Source data are provided as a Source Data file.

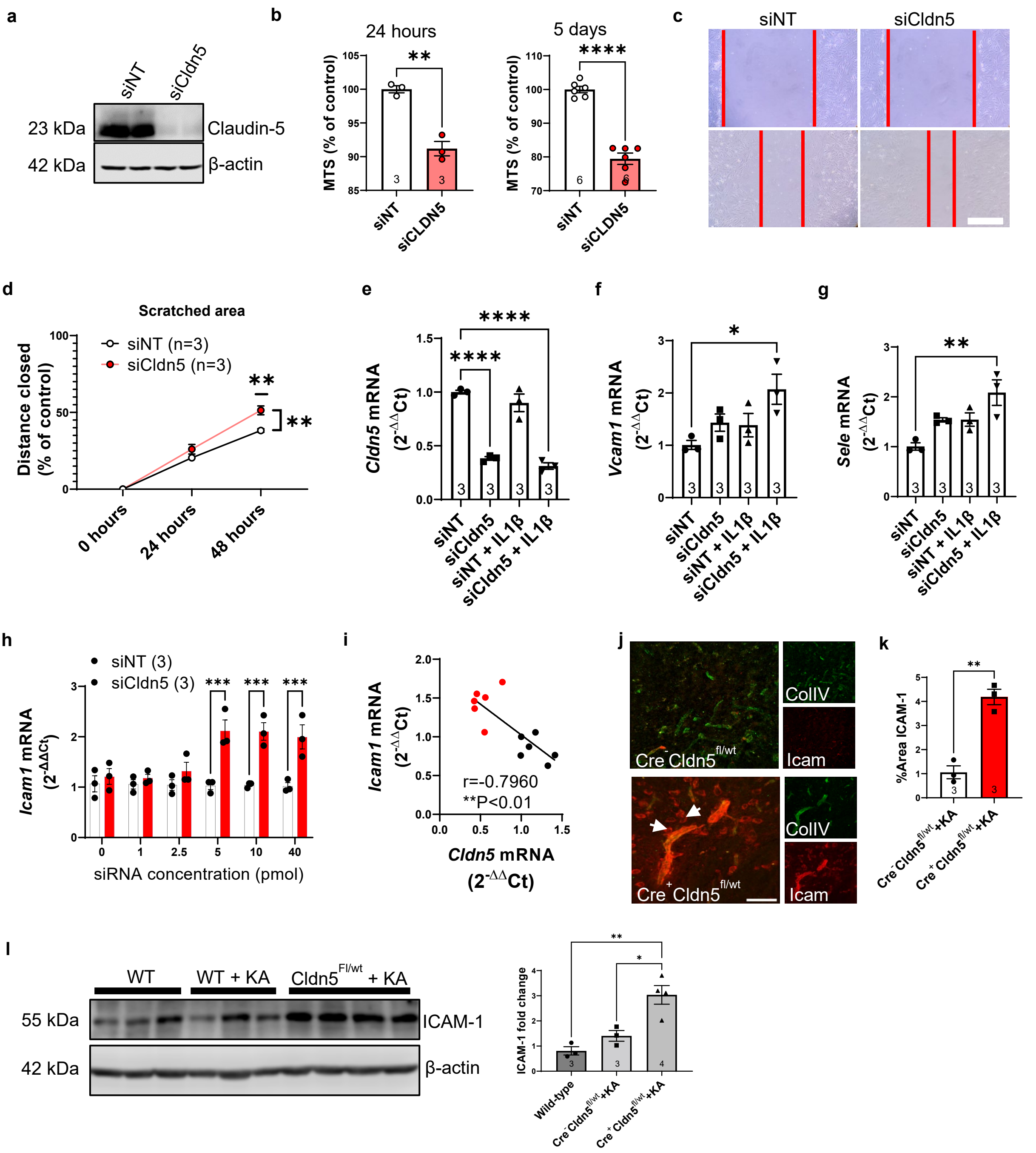

Supplementary Figure 6. Knockdown of claudin-5 in endothelial cells induces expression of cell adhesion molecules and increases immune cell infiltration. a) Suppression of claudin-5 post transfection of claudin-5 targeting siRNA (siCldn5) compared to non-targeting (siNT) transfected cells. Western blot is representative of 3 independent experiments. **b)** Decreased MTS bio reduction post suppression of claudin-5 at 24 h (**p=0.0018) and 5 days (****p<0.0001). **c) & d)** Increased wound closure 48 hours post transfection of siCldn5

(**p=0.0012). Scale bar, 200 μm. **e)** Decreased *Cldn5* transcript in siNT or siCldn5 transfected bEnd.3 cells post-stimulation with 10 ng/ml IL-1β for 24 hours (****p<0.0001). Increased **f)** *Vcam1* (*p=0.0157) and **g)** *Sele* (**p=0.0025) mRNA expression. **h)** Increased mRNA expression of *Icam1* following 5 nM (***p=0.0001), 10 nM (***p=0.0001) and 40 nM (***p=0.0004) siRNA-mediated suppression of *Cldn5* in bEnd.3 cells. **i)** Significant correlation between *Icam1* and *Cldn5* (r=-0.7960; **p=0.002). **j)** Increased Icam1 expression in $Cre^{+}Cldn5^{fl/wt}$ mice following i.p. injection of kainic acid (KA). Scale bar, 50 μm. **k)** Quantification of Icam1 expression (**p=0.0017). **l)** Western blot analysis of Icam1 revealed increased expression 48 hours following i.p. injection of 15 mg/kg KA in $Cre^{+}Cldn5^{fl/wt}$ mice compared to $Cre^{-}Cldn5^{fl/wt}$ (*p=0.0141). Data represent means ± s.e.m.; each datapoint represents one independent experiment or one animal. Number of independent experiments or animals (n) is indicated on graphs. Two-way ANOVA followed by Bonferroni's multiple comparison test for scratch assay and siRNA experiment; two-sided unpaired *t*-test for MTS assay; one-way ANOVA followed by Tukey's multiple comparison test for all other graphs. *p<0.05; **p<0.01; ***p<0.001; ****p<0.0001. Source data are provided as a Source Data file.

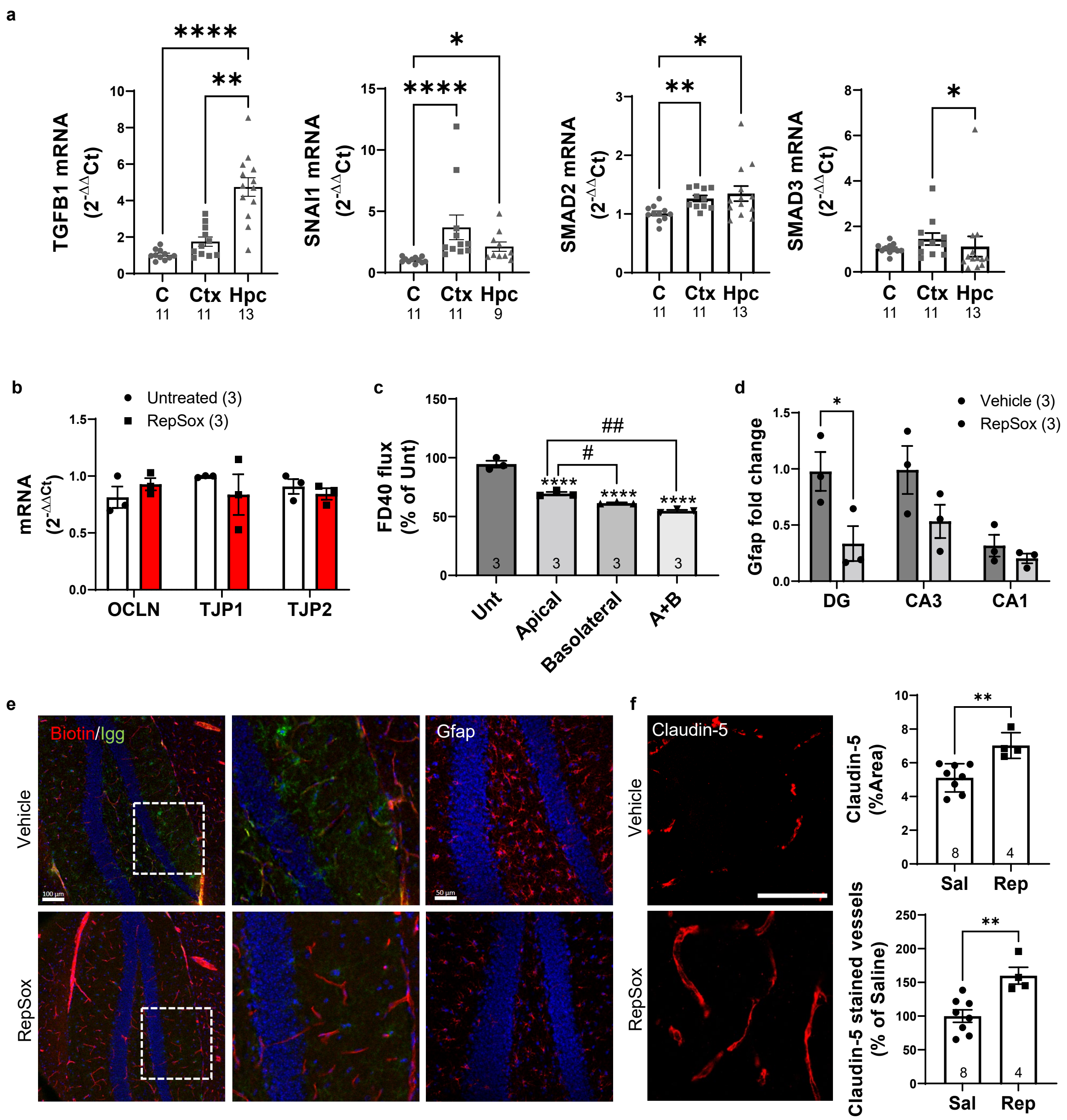

Supplementary Figure 7. RepSox rescues kainic acid-induced BBB leakiness and claudin-5 downregulation. a) TGFB1 (****p<0.0001), SNAI1 (****p<0.0001 for cortex (Ctx) vs control (C); *p=0.0141 for hippocampus (Hpc) vs C) and SMAD2 (**p=0.0082 for Ctx vs C; *p=0.0381 for Hpc vs C) expression were all increased in temporal lobe epilepsy (TLE) brain tissue compared to autopsy control brain. **b**) No change in expression of OCLN, TJP1, TJP2 in

hCMEC/d3 cells 24 hours following treatment with 100 μM RepSox. **c**) 40 kDa FITC-dextran (FD40) flux in hCMEC/d3 cells was significantly reduced 24 hours following treatment with 100 μM RepSox on the apical, basolateral or apical+basolateral (A+B) side (****p<0.0001 for all) compared to untreated (Unt). **d)** GFAP expression was significantly reduced in the dentate gyrus (DG) (*p=0.0299) in mice injected with vehicle or 10 mg/kg RepSox 7 days post intrahippocampal injection of 0.3 μg kainic acid. **e)** Biotin, IgG and GFAP staining in mice injected with vehicle or 10 mg/kg RepSox 7 days post intrahippocampal injection of 0.3 μg kainic acid. Images are representative of n=3 animals per group. Scale bars, 100 μm for Biotin/IgG; 50 μm for GFAP. **f)** Claudin-5 staining, and analysis revealed increased claudin-5-stained area (**p=0.0032) and claudin-5 vessel length (**p=0.0035) in RepSox (Rep) injected compared to saline (Sal) injected mice. Scale bar, 50 μm. Data represent means ± s.e.m; each data point represents one subject, one independent experiment or one animal. Number of subjects/experiments (n) is indicated on graphs. Two-sided unpaired *t*-test for Racine score and claudin-5 immunohistochemistry; one-way ANOVA followed by Tukey's multiple comparison test for FD40 flux assay; two-way ANOVA followed by Bonferroni's multiple comparison test for GFAP quantification; Kruskal-Wallis test followed by Dunn's multiple comparison test for human gene expression data. *p<0.05; **p<0.01; ****p<0.0001. Source data are provided as a Source Data file.

Supplementary Table 1. Demographic details of temporal lobe epilepsy cases

Abbreviations: TLE: temporal lobe epilepsy; FA: focal aware; FIA: focal impaired aware; FBTCS: focal to bilateral tonic-clonic seizure; HS: hippocampal sclerosis; yrs: years.

Average age: 43.7

Number male/female: 9/7

#	Type epilepsy	Type seizure	Seizure frequency	Duration of epilepsy (yrs)	Pathology
1	TLE (L)	2 FIA	Monthly	5	Minimal cortical dysplasia
2	TLE (L)	2 FIA, 3 FBTCS	>1/week	40	HS (severe)
3	Extra-TLE (R)	2 FIA	>1/day	20	Normal
4	TLE (R)	2 FIA, 3 FBTCS	Monthly	25	Oligodendrocyte hyperplasia
5	TLE (L)	2 FIA	Monthly	14	Normal
6	TLE (L)	2 FIA	>1/month	7	Subpial gliosis, mild oligodendrocyte hyperplasia, heterotopic white matter, HCC evaluation not possible
7	TLE (bi-temporal R>L)	2 FIA, 3 FBTCS	>1/day	62	Subpial gliosis, mild oligodendrocyte hyperplasia, HCC granular neuron dispersion

8	TLE (L)	2 FIA, 3 FBTCS	>1/day	38	Subpial gliosis
9	TLE (L)	2FIA, 3FBTCS	>1/week	32	Subpial gliosis
10	TLE (L)	2FIA, 3FBTCS	>1/month	17	Increased subcortical oligodendroglial like cells
11	TLE (L)	2 FIA	Monthly	8	Oligodendrocyte hyperplasia, HS
12	TLE (R)	1FA, 3 FBTCS	Daily	9	Subpial gliosis, HS
13	TLE (L)	2 FIA	>1/month	38	Mild pyramidial neuronal loss
14	TLE (L)	1 FA, 2 FIA, 3 FBTCS	>1/month	24	Chaslins subpial gliosis
15	TLE (L)	1 FA, 2 FIA, 3 FBTCS	>1/week	8	Gliosis, neuronal depletion + dispersion, hippocampal dentate fascia + marked neuronal loss CAI
16	TLE (L)	1 FA, 2 FIA, 3 FBTCS	>1/month	9	Chaslin sub pial gliosis

Supplementary Table 2. Demographic details of control cases

Average age: 46.7

Number male/female: 6/5

#	Age at death	Post-mortem Interval (hours)
C1	57	26
C2	29	42
C3	58	27
C4	68	13
C5	35	40
C6	42	27
C7	41	11
C8	35	23
C9	44	10
C10	53	28
C11	52	22

Supplementary Table 3. qPCR primers

Mouse	Forward primer (5'-3')	Reverse primer (5'-3')
Cldn5	TTTCTTCTATGCGCAGTTGG	GCAGTTTGGTGCCTACTTCA
Ocln	ACAGTCCAATGGCCTACTCC	ACTTCAGGCACCAGAGGTGT
Tjp1	CCACCTCTGTCCAGCTCTTC	CACCGGAGTGATGGTTTTCT
Marveld2	AACCCCCTTACAGCTGTCCT	TAATCCCGTCAGCATCTTCC
Jam2	TGGTGGAGCTACGATGCCAG	AGCTGTTGTTGTGTGTGCCG
Icam1	TGTCAGCCACCATGCCTTAG	CAGCTTGCACGACCCTTCTA
Vcam1	GAAGCCGGTCACAGTCAAGT	CCTCGCTGGAACAGGTCATT
Sele	GCTACCCATGGAACACGACA	CTTTGCATGATGGCGTCTCG
Fos	GGAATTAACCTGGTGCTGGA	CATTCAGACCACCTCGACAA
Actab	TCACCCACACTGTGCCCATCTACGA	CAGCGGAACCGCTCATTGCCAATGG
Il1b	GCCACCTTTTGACAGTGATGAG	AAGGTCCACGGGAAAGACAC
**Human**	**Forward primer (5'-3')**	**Reverse primer (5'-3')**
CLDN5	CTGGACCACAACATCGTGA	CACCGAGTCGTACACTTTGC
OCLN	TCAGGGAATATCCACCTATCACTTCAG	CATCAGCAGCAGCCATGTACTCTTCAC
TJP1	CGGTCCTCTGAGCCTGTAAG	GGATCTACATGCGACGACAA
TJP2	ATCAGACTAGCCACTCCTGC	AACCCAGTCCCACAAACAGA
SNAI1	GAGGACAGTGGGAAAGGCTC	TGGCTTCGGATGTGCATCTT
SMAD2	ATGTCGTCCATCTTGCCATTC	AACCGTCCTGTTTTCTTTAGCTT
SMAD3	CGCATGAGCTTCGTCAAAGG	AGAGTCACCTGGAGTTGGGT
GFAP	GCACCAAAGACGGGGAAAAT	CTTTAGGGGAAGCCTGGGAA
