# Supplementary material for: Microvascular stabilization via blood-brain barrier regulation prevents seizure activity
Source: Nat Commun. 2022 Apr 14;13:2003. doi: 10.1038/s41467-022-29657-y (PMC9010415; doi:10.1038/s41467-022-29657-y)
Supplement: Supplementary file 3 — Reporting Summary [file 41467_2022_29657_MOESM3_ESM.pdf]

nature portfolio

Corresponding author(s): Matthew Campbell

Last updated by author(s): Feb 05, 2022

Reporting Summary

Nature Portfolio wishes to improve the reproducibility of the work that we publish. This form provides structure for consistency and transparency in reporting. For further information on Nature Portfolio policies, see our Editorial Policies and the Editorial Policy Checklist.

Statistics

For all statistical analyses, confirm that the following items are present in the figure legend, table legend, main text, or Methods section.

n/a	Confirmed	
☐	☒	The exact sample size (*n*) for each experimental group/condition, given as a discrete number and unit of measurement
☐	☒	A statement on whether measurements were taken from distinct samples or whether the same sample was measured repeatedly
☐	☒	The statistical test(s) used AND whether they are one- or two-sided<br>*Only common tests should be described solely by name; describe more complex techniques in the Methods section.*
☐	☒	A description of all covariates tested
☐	☒	A description of any assumptions or corrections, such as tests of normality and adjustment for multiple comparisons
☐	☒	A full description of the statistical parameters including central tendency (e.g. means) or other basic estimates (e.g. regression coefficient) AND variation (e.g. standard deviation) or associated estimates of uncertainty (e.g. confidence intervals)
☐	☒	For null hypothesis testing, the test statistic (e.g. *F*, *t*, *r*) with confidence intervals, effect sizes, degrees of freedom and *P* value noted<br>*Give P values as exact values whenever suitable.*
☒	☐	For Bayesian analysis, information on the choice of priors and Markov chain Monte Carlo settings
☒	☐	For hierarchical and complex designs, identification of the appropriate level for tests and full reporting of outcomes
☐	☒	Estimates of effect sizes (e.g. Cohen's *d*, Pearson's *r*), indicating how they were calculated

Our web collection on statistics for biologists contains articles on many of the points above.

Software and code

Policy information about availability of computer code

Data collection: Where possible, behavioural tests were recorded and analysed Using AnyMaze software (AnyMaze 4.99m Stoelting Co.). EEG data was collected through Natus Neurowork

Data analysis: Data analysis was performed in GraphPad Prism software (version 9.0). Normality was determined by D'Agostino-Pearson, Shapiro-Wilk and Kolmogorov-Smirnov tests. Most datasets were normally distributed and then t-tests, ANOVAs and Pearson's correlations were performed. Bonferroni or Tukey post-hoc tests were performed where appropriate for one-way and two-way ANOVAs with statistical significance set at P<0.05. Confocal images and western blots were analysed with ImageJ v1.53f. LabChart 8 was used for quantification of EEG data. MATLAB R2020b was used for DCE image generation.

For manuscripts utilizing custom algorithms or software that are central to the research but not yet described in published literature, software must be made available to editors and reviewers. We strongly encourage code deposition in a community repository (e.g. GitHub). See the Nature Portfolio guidelines for submitting code & software for further information.

Data

Policy information about availability of data

All manuscripts must include a data availability statement. This statement should provide the following information, where applicable:

- Accession codes, unique identifiers, or web links for publicly available datasets
- A description of any restrictions on data availability
- For clinical datasets or third party data, please ensure that the statement adheres to our policy

There are no restrictions on data availability in our manuscript. There are no large datasets that require web links. All data supporting the findings of this study are available within the paper and Supplementary Information files.

Field-specific reporting

Please select the one below that is the best fit for your research. If you are not sure, read the appropriate sections before making your selection.

☒ Life sciences ☐ Behavioural & social sciences ☐ Ecological, evolutionary & environmental sciences

For a reference copy of the document with all sections, see nature.com/documents/nr-reporting-summary-flat.pdf

Life sciences study design

All studies must disclose on these points even when the disclosure is negative.

Sample size	G*Power was used to determine sample sizes for all experiments.
Data exclusions	Outliers for behavioral testing were identified as being greater than two standard deviations from the mean and excluded from statistical analysis.
Replication	All experiments were replicated in triplicate.
Randomization	Animals were randomized for experiments and littermates were used in all experimental groups and across treatment groups. For viral injections, kainic acid injections and RepSox injections, mice were randomly assigned to control or experimental groups. For human tissue experiments, resected brain tissue from pharmacoresistant epilepsy patients were provided by Beaumont Hospital. Similar numbers of male/female samples were obtained and control tissue obtained from the Stanley Medical Research Foundation contained equal numbers of male/female. Covariates were not controlled. For cell culture experiments, wells were randomly assigned treatments.
Blinding	All in vivo work related to phenotyping seizure activity in mice was performed by an individual blinded to the experiment. For behavioural experiments, automated tracking software was used or if not available, an experimenter blind to treatment scored behaviour. For immunohistochemistry and quantification, an experimenter blind to treatment groups and IDs performed analysis and groups were revealed following analysis.

Reporting for specific materials, systems and methods

We require information from authors about some types of materials, experimental systems and methods used in many studies. Here, indicate whether each material, system or method listed is relevant to your study. If you are not sure if a list item applies to your research, read the appropriate section before selecting a response.

Materials & experimental systems

n/a	Involved in the study	
☐	☒	Antibodies
☐	☒	Eukaryotic cell lines
☒	☐	Palaeontology and archaeology
☐	☒	Animals and other organisms
☐	☒	Human research participants
☐	☒	Clinical data
☒	☐	Dual use research of concern

Methods

n/a	Involved in the study	
☒	☐	ChIP-seq
☒	☐	Flow cytometry
☐	☑	MRI-based neuroimaging

Antibodies

Antibodies used	Primary antibodies used were rabbit anti-claudin-5 (1/500 Life Technologies, #34-1600), rabbit anti-CD31 (1/100 Abcam, #ab28364), rat anti-CD31 (1/100 BD Biosciences, #550274) rabbit anti- IBA1 (1/500 Wako, #019-19741), rabbit anti-human fibrinogen FITC (1/100 DAKO, #F0111) , goat anti-human IgG Cy3 (1/100 Abcam, #ab97170), mouse anti-GFAP (1/500 Sigma, #G3893), isolectin GS-B4 Alexa Fluor 568 (1/300 Biosciences, #I21412), goat anti-rabbit IgG Alexa Fluor 488 (1/500 Abcam, #ab150077), goat anti-rat Alexa Fluor 594 (1/500 Abcam, #ab150160).
Validation	Primary antibodies were used according to instructions on the manufacturers website and according to the following references: rabbit anti-claudin-5, rat anti-CD31, rabbit anti-human fibrinogen, isolectin GS-B4 Alexa Fluor 568, goat anti-rabbit IgG Alexa Fluor 488 and goat anti-rat Alexa Fluor 594 (Greene et al., 2017, Menard et al., 2017, Keaney et al., 2015).

Eukaryotic cell lines

Policy information about cell lines

Cell line source(s)	bEnd.3 cells were sourced from ATCC. hCMEC/d3 cells were sourced from Merck Millipore
Authentication	CD31 staining was used to confirm endothelial cells in hCMEC and b.End3 cell lines.
Mycoplasma contamination	All cells were negative for mycoplasma
Commonly misidentified lines (See ICLAC register)	No commonly mididentified cell lines were used in the study

Palaeontology and Archaeology

Specimen provenance	n/a
Specimen deposition	n/a
Dating methods	n./a

☐ Tick this box to confirm that the raw and calibrated dates are available in the paper or in Supplementary Information.

Ethics oversight	n/a

Note that full information on the approval of the study protocol must also be provided in the manuscript.

Animals and other organisms

Policy information about studies involving animals; ARRIVE guidelines recommended for reporting animal research

Laboratory animals	Male C57BL/6J, Cldn5 fl/fl mice and Cldn5 160 mice (~20 g) were bred on-site and used at 8-12 weeks of age. For EEG experiments, all mice were singly house following electrode implantation. All mice were maintained on a 12 h/12 h light/dark cycle. Room temperature was maintained between 18 and 23C and humidity kept at 40-5 %. Mice were provided with ad libitum access to food and water.
Wild animals	No wild animals were used in the study
Field-collected samples	No field collected samples were used in the study.
Ethics oversight	The Research Ethics Committees of Trinity College Dublin and RCSI approved the protocols and procedures used in this study under licence from the Department of Health (HPRA AE19136/PO80 and AE19127/PO57

Note that full information on the approval of the study protocol must also be provided in the manuscript.

Human research participants

Policy information about studies involving human research participants

Population characteristics	All demographic information including age, number of each sex, diagnosis, postmortem interval of human participants is included in the Supplementary Tables 1 and 2. MRI, EEG and treatment regimen was used to diagnose patients with refractory temporal lobe epilepsy. Autopsy control cohorts from the Stanley Medical Research Institute were matched as closely as possible for sex and age. Additionally, non-epileptic tissue obtained during surgical resection was used for comparisons.
Recruitment	All participants recruited through the St James's Hospital epilepsy clinic run by Prof Colin Doherty (co-author) were diagnosed with drug-resistant temporal lobe epilepsy.
Ethics oversight	Ethical approval was obtained through the St James's Hospital ethics committee.

Note that full information on the approval of the study protocol must also be provided in the manuscript.

Clinical data

Policy information about clinical studies

All manuscripts should comply with the ICMJE guidelines for publication of clinical research and a completed CONSORT checklist must be included with all submissions.

Clinical trial registration	n/a
Study protocol	n/a
Data collection	All data was collected in St James's Hospital Dublin, Ireland.
Outcomes	n/a

Dual use research of concern

Policy information about dual use research of concern

Hazards

Could the accidental, deliberate or reckless misuse of agents or technologies generated in the work, or the application of information presented in the manuscript, pose a threat to:

No	Yes	
☒	☐	Public health
☒	☐	National security
☒	☐	Crops and/or livestock
☒	☐	Ecosystems
☒	☐	Any other significant area

Experiments of concern

Does the work involve any of these experiments of concern:

No	Yes	
☒	☐	Demonstrate how to render a vaccine ineffective
☒	☐	Confer resistance to therapeutically useful antibiotics or antiviral agents
☒	☐	Enhance the virulence of a pathogen or render a nonpathogen virulent
☒	☐	Increase transmissibility of a pathogen
☒	☐	Alter the host range of a pathogen
☒	☐	Enable evasion of diagnostic/detection modalities
☒	☐	Enable the weaponization of a biological agent or toxin
☒	☐	Any other potentially harmful combination of experiments and agents

ChIP-seq

Data deposition

☐ Confirm that both raw and final processed data have been deposited in a public database such as GEO.

☐ Confirm that you have deposited or provided access to graph files (e.g. BED files) for the called peaks.

Data access links<br>*May remain private before publication.*	n/a
Files in database submission	n/a
Genome browser session<br>(e.g. UCSC)	n/a

Methodology

Replicates	n/a
Sequencing depth	n/a
Antibodies	n/a
Peak calling parameters	n/a
Data quality	n/a
Software	n/a

Flow Cytometry

Plots

Confirm that:

☐ The axis labels state the marker and fluorochrome used (e.g. CD4-FITC).

☐ The axis scales are clearly visible. Include numbers along axes only for bottom left plot of group (a 'group' is an analysis of identical markers).

☐ All plots are contour plots with outliers or pseudocolor plots.

☐ A numerical value for number of cells or percentage (with statistics) is provided.

Methodology

Sample preparation | *Describe the sample preparation, detailing the biological source of the cells and any tissue processing steps used.*

Instrument | *Identify the instrument used for data collection, specifying make and model number.*

Software | *Describe the software used to collect and analyze the flow cytometry data. For custom code that has been deposited into a community repository, provide accession details.*

Cell population abundance | *Describe the abundance of the relevant cell populations within post-sort fractions, providing details on the purity of the samples and how it was determined.*

Gating strategy | *Describe the gating strategy used for all relevant experiments, specifying the preliminary FSC/SSC gates of the starting cell population, indicating where boundaries between "positive" and "negative" staining cell populations are defined.*

☐ Tick this box to confirm that a figure exemplifying the gating strategy is provided in the Supplementary Information.

Magnetic resonance imaging

Experimental design

Design type: Dynamic Contrast enhanced MRI

Design specifications: We used gadolinium based contrast agent for (DCE-MRI) scans

Behavioral performance measures: n/a

Acquisition

Imaging type(s): DCE-MRI

Field strength: 3T

Sequence & imaging parameters: DCE-MRI using T1 weighted imaging.

Area of acquisition: Whole brain

Diffusion MRI ☐ Used ☒ Not used

Preprocessing

Preprocessing software: We measured gadolinium extravasation using KTrans.

Normalization: n/a

Normalization template: n/a

Noise and artifact removal: n/a

Volume censoring: n/a

Statistical modeling & inference

Model type and settings: DCE-MRI (KTrans measurements)

Effect(s) tested: n/a

Specify type of analysis: ☒ Whole brain ☐ ROI-based ☐ Both

Statistic type for inference (See Eklund et al. 2016): n/a

Correction: n/a

Models & analysis

n/a	Involved in the study
☒	☐ Functional and/or effective connectivity
☒	☐ Graph analysis
☒	☐ Multivariate modeling or predictive analysis
